# Research progress on m6A and drug resistance in gastrointestinal tumors

**DOI:** 10.3389/fphar.2025.1565738

**Published:** 2025-04-28

**Authors:** Ziyi Xu, Bo Sun, Weizheng Wang, Yitao Fan, Jingxiang Su, Jiachun Sun, Xinyu Gu

**Affiliations:** Henan Key Laboratory of Cancer Epigenetics, Cancer Institute, The First Affiliated Hospital, College of Clinical Medicine, Medical College of Henan University of Science and Technology, Luoyang, China

**Keywords:** m6A, RNA modifications, drug resistance, gastrointestinal tumors, epigenetic alterations

## Abstract

Gastrointestinal (GI) tumors represent a significant global health burden and are among the leading causes of cancer-related mortality worldwide. their drug resistance is one of the major challenges in cancer therapy. In recent years, epigenetic modifications, especially N6-methyladenosine (m6A) RNA modifications, have become a hot research topic. m6A modification plays an important role in gene expression and cancer progression by regulating RNA splicing, translation, stability, and degradation, which are regulated by “writers,” “erasers” and “readers.” In GI tumors, resistance to chemotherapy, targeted therapy, and immunotherapy is closely associated with m6A RNA modification. Therefore, the molecular mechanism of m6A modification and its targeted drug development provide new therapeutic strategies for overcoming drug resistance and therapeutic efficacy in GI tumors. In this review, the biological functions of m6A were explored, the specific resistance mechanisms of m6A in different types of GI tumors were explored, new ideas and targets for future treatment resistance were identified, and the limitations of this field were highlighted.

## 1 Introduction

GI tumors, including esophageal cancer (EC), gastric cancer (GC), hepatic cancer (HCC), colorectal cancer (CRC), and pancreatic cancer (PC) (Vedeld et al., 2018; Liu Y. et al., 2023), are among the most prevalent and deadly malignant tumors worldwide (Turkes et al., 2020; Tang et al., 2022; Zhao et al., 2024). According to the Global Cancer Statistics 2020 (GLOBOCAN) report, gastrointestinal tumors account for more than 4 million new cases and nearly 3 million deaths annually, thereby constituting a significant component of the global cancer burden (Sung et al., 2021). Although radiotherapy, chemotherapy, targeted therapy, and immunotherapy have become the dominant cancer treatments (Tannock, 2001; Dagogo-Jack and Shaw, 2018; Li G. H. et al., 2021), patients with advanced or metastatic forms of the disease often respond poorly to these therapies (Tannock, 2001; Li et al., 2024). Moreover, drug resistance in cancer treatment is one of the greatest challenges currently faced and is a major factor limiting the potential for patient recovery (Viswanathan et al., 2017; Saw et al., 2023). Drug resistance includes intrinsic resistance (present at the start of treatment) and acquired resistance (occurring during or after initial treatment) (Akiyama et al., 2023; Che et al., 2024; Dong et al., 2024). The mechanism of tumor drug resistance is very complex and mainly involves enhanced DNA damage repair, gene mutation (Rosenthal et al., 2019), the tumor microenvironment (TME) (Akiyama et al., 2023; Liu N. et al., 2023; Pan W. et al., 2024), and the overexpression of drug efflux pumps (Lin C. et al., 2022; Zhuang et al., 2023).

**FIGURE 1:**
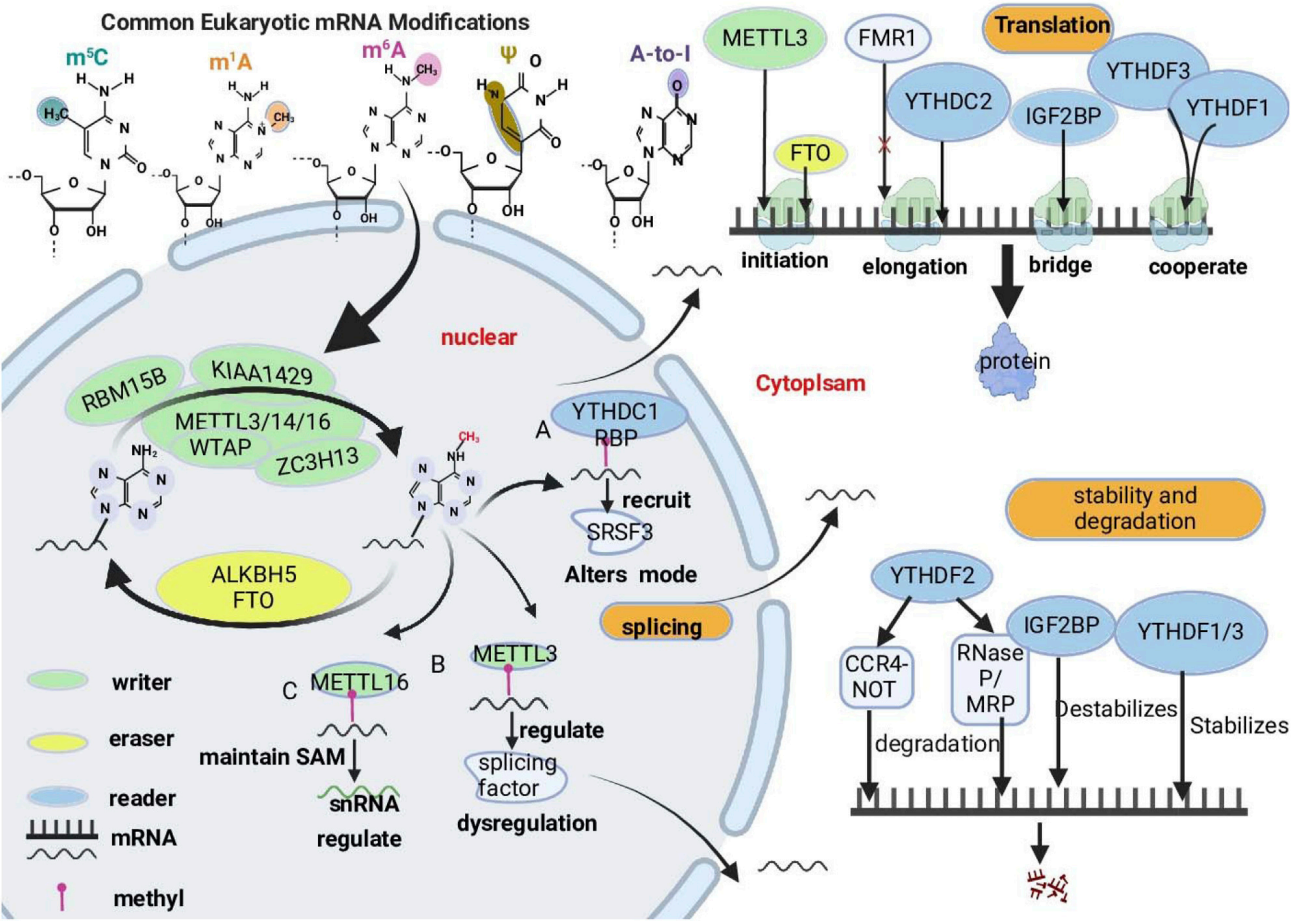
Overview of key RNA modifications closely linked to cancer progression. The major types of RNA modifications, including 5-methylcytosine (m5C), N1-methyladenosine (m1A), N6-methyladenosine (m6A), pseudouridine (Ψ), and adenosine-to-inosine (A-to-I), focus on the roles of m6A in regulating RNA metabolism. These modifications alter RNA structure and function, impacting key biological processes such as splicing, translation, and stability and degradation. RMs are dynamic and often reversible. RMs are dynamically regulated by “writers,” “readers,” and “erasers.” “Writer” proteins are responsible for transferring methyl groups onto RNA molecules. (e.g., METTL3, METTL14, WTAP and ZC3H13), “readers” recognize and bind to m6A-modified RNA molecules. (e.g., YTHDF proteins, IGF2BPs), and “erasers” remove RNA modifications (e.g., FTO and ALKBH5). The complex interactions among writers, readers, and erasers are critical for cellular adaptations and responses, especially in the context of cancer.

In addition to the above factors, epigenetic modifications, which can alter gene expression without altering the DNA sequence, are also closely associated with drug resistance (Nagaraju et al., 2021; Tang B. et al., 2023). Common epigenetic modifications in cancer include DNA modifications, histone modifications, RNA modifications, and chromatin remodeling. These mechanisms play important roles in regulating gene expression, cell fate decisions, and response to environmental stimuli, and the occurrence of epigenetic abnormalities is often an important driver of cancer development (Arrowsmith et al., 2012; An and Duan, 2022). Among these modifications, RNA modifications have become a focal point of research. Various types of RNA (Frye et al., 2018; Chellamuthu and Gray, 2020; Jin et al., 2021), including messenger RNAs (mRNAs), transfer RNAs (tRNAs), ribosomal RNAs (rRNAs), and long noncoding RNAs (lncRNAs), are modified (Roundtree et al., 2017; Ontiveros et al., 2019; Huang et al., 2020). Similar to modifications of DNA and histones, RNA modifications are mediated by specific methyltransferases (“Writers”) and demethylases (“Erasers”), whose functions are determined by recognition proteins (“Reader”) (Zaccara et al., 2019; Sarraf and Chhabra, 2023; Chen D. et al., 2024). The main types of RNA modifications include N6-methyladenosine (m6A), N1-methyladenosine (m1A), 5-methylcytosine (m5C), inosine (I), and pseudouridine (Ψ) modifications (Liu and Gregory, 2019), among which m6A is the most abundant RNA modification (Shi et al., 2019; Yue et al., 2019; An and Duan, 2022) (Figure 1). m6A RNA modification, characterized by its reversible and dynamic nature, determines the fate of modified RNA molecules after transcription (Wang Q. et al., 2020) and also regulates RNA transcription prior to splicing. The exon junction complex (EJC) has been identified as an inhibitor of m6A modification, spatially controlling the deposition of m6A modifications, thereby influencing RNA transcription (Yang et al., 2022; He et al., 2023). Research indicates that m6A modifications on chromatin-associated regulatory RNAs (caRNAs) can globally regulate chromatin status and transcription. In mouse embryonic stem cells, the depletion of m6A methylation-related enzymes METTL3 or YTHDC1, in an m6A-dependent manner via carRNAs, increases chromatin accessibility and promotes transcription (Liu et al., 2020). Furthermore, m6A also directly affecting gene expression and cellular function by influencing RNA splicing, translation, and stability (Liu et al., 2017; Wang T. et al., 2020). In cancer, aberrant m6A modifications are closely associated with tumorigenesis, invasion, proliferation, and drug resistance, which influences the effectiveness of tumor therapies (Nombela et al., 2021). For example, overexpression of the methyltransferase METTL3 has been shown to increase the stability of oncogenes and promote tumor growth (Wang Q. et al., 2020), whereas the demethylase FTO induces m6A demethylation to increase the expression of multidrug resistance genes (Yang et al., 2019).

Increasing evidence suggests that m6A has dual functions in cancer. m6A influences tumor progression by regulating oncogene or tumor suppressor gene expression. Additionally, the levels of m6A and the expression or activity of its enzymes can also be modulated (Li H. et al., 2021; Niu et al., 2022). This review summarizes the latest research on RNA modifications, particularly the role of m6A in cancer treatment resistance. In addition, it provides insights into the biological functions of m6A. Finally, we explore the specific resistance mechanisms of m6A in different types of GI tumors, which will provide new ideas and targets for the future of therapeutic resistance.

## 2 Biological functions of m6A RNA modifications

m6A modification is an RNA modification that is widely present in eukaryotes and is particularly notable in human mRNAs and ncRNAs(Wang et al., 2014; Barbieri and Kouzarides, 2020). m6A is a methylation that occurs at the nitrogen atom at the 6th position of adenosine (Meyer et al., 2012; He et al., 2019). It primarily exists in the RACH sequence; is enriched at the 3′-UTR near the stop codon, as well as in long introns and exons (He and He, 2021); and is regulated by specific writers, erasers, and readers (Chen et al., 2019). The methyltransferase complex (MTC) consists of methyltransferase-like protein 3 (METTL3), methyltransferase-like protein 14 (METTL14), methyltransferase-like protein 16 (METTL16), zinc finger CCCH-type containing 13 (ZC3H13) (Gong et al., 2020; Zhao et al., 2021), RNA-binding motif protein 15 (RBM15), RNA-binding motif protein 15B (RBM15B) (Wang Q. et al., 2021), Wilms tumor 1-associated protein (WTAP), and virus-like m6A methylation-associated protein (VIRMA, also known as KIAA1429) (Li R. et al., 2021), which are responsible for transferring methyl groups from the donor S-adenosylmethionine (SAM) to the nitrogen atom in the sixth position of adenosine (Kagan and Clarke, 1994; Salyan et al., 2006). METTL3, METTL14, and WTAP are core members of the complex (Jiang et al., 2021; Li G. H. et al., 2021; Oerum et al., 2021); one of these, METTL3, is the catalytic core of the m6A MTC, and other proteins support the complex (Sendinc and Shi, 2023). In the nucleus, m6A demethylases can reverse m6A methylation, and the demethylases include AlkB homolog 5 (ALKBH5) and obesity-associated protein (FTO) (Jia et al., 2011). m6A methylation is recognized by binding proteins such as the YTH structural domain family of proteins (YTHDF proteins, including YTHDF1, YTHDF2, YTHDF3, YTHDC1, and YTHDC2) (Wang S. et al., 2021; Yen and Chen, 2021; Xu et al., 2023), insulin-like growth factor 2 (IGF2BP) mRNA-binding proteins (Zeng et al., 2025), heterogeneous nuclear ribonucleoprotein (HNRNP) family of proteins, and eukaryotic translation initiation factor 3 (eIF3) proteins (Niu et al., 2022). Dynamic changes in these three regulatory proteins play significant roles in regulating gene expression at the posttranscriptional level, including processes such as splicing, translation, RNA degradation, and stability, which are involved in the occurrence and metastasis of a variety of malignant tumors and may be promising targets for anticancer therapy (Boulias and Greer, 2023; Deng et al., 2023; Zhu L. et al., 2023). Therefore, through the in-depth study of m6A modifications and their biological functions, we can provide new strategies for the treatment of GI digestive tract tumors (Dolcetti et al., 2018) (Figure 1) (Table 1).

**TABLE 1:** The biological functions of m6A.

Biological function	Gene	Type	Mechanism	References
splicing	YTHDC1	reader	Recruits SRSF3 to modulate RNA splicing patterns	Xiao et al. (2016), Kasowitz et al. (2018)
	METTL3	writer	Modulates splicing factors, inducing splicing dysregulation	Wu et al. (2023)
	METTL16	writer	Maintains the intracellular homeostasis of SAM, Participating in the modification of snRNAs, influencing pre-mRNA splicing	Xiao et al. (2016)
	RBP	reader	Influencing the recruitment of splicing factors, altering splicing patterns	Zhu et al. (2023a)
Translation	eIF3	reader	Recruits the 43S ribosomal complex to facilitate cap-independent translation initiation	Zhang et al. (2018)
	YTHDF1	reader	Enhances translation efficiency	Wang et al. (2015), Sendinc and Shi (2023)
	YTHDC2	reader	Promotes the binding of mRNA to ribosomes	Kretschmer et al. (2018)
	YTHDF3	reader	Interacts with YTHDF1 to enhance translation efficiency	Shi et al. (2017)
	METTL3	writer	Interacts with eIF3	Choe et al. (2018), Shi et al. (2019)
	IGF2BPs	reader	Stabilizes mRNA	Huang et al. (2018)
	FMR1	RNA binding protein	Inhibits ribosome binding and translation	Edupuganti et al. (2017)
Stability and degradation	YTHDF2	reader	Interacts with the CCR4-NOT complex and the RNase P/MRP complex	Collart, 2016; Du et al. (2016), Zhao et al. (2017), Park et al. (2019), Boo and Kim (2020)
	YTHDF1/YTHDF3	reader	Destabilizes mRNA	Hsu et al. (2017)
	IGF2BPs	reader	Stabilizes mRNA by preventing its degradation	Huang et al. (2018)

### 2.1 RNA splicing

RNA splicing plays a significant role in the gene expression process of eukaryotes, which involves exercising noncoding regions, introns, and long noncoding RNAs (lncRNAs) from precursor messenger RNAs (pre-mRNAs) and then joining exons to form mature mRNAs, serving as a critical step in the conversion of genetic information from DNA to proteins (Shi, 2017; Montes et al., 2019).

Studies have shown that m6A modification can directly prevent the binding of the splicing factor U2 auxiliary factor (U2AF) 35 (U2AF35) to pre-mRNA. U2AF35 is a subunit of the U2AF heterodimer that directly binds to the AG dinucleotide at the 3′splice site and is a key step in splicing initiation (Nombela et al., 2021). The presence of the m6A modification reduces the affinity of U2AF35 for RNA, thereby inhibiting correct splicing of pre-mRNA (Mendel et al., 2021). Evidence suggests that YTHDC1 binds to m6A-modified regions and recruits serine/arginine-rich splicing factor 3 (SRSF3), thereby altering the splicing pattern of mRNAs (Xiao et al., 2016; Kasowitz et al., 2018). METTL3 induces the upregulation of splicing factors, whereas METTL3 deficiency affects the translation of splicing factors, which leads to splicing dysregulation and affects cancer development (Wu et al., 2023). METTL16 maintains intracellular SAM homeostasis and is involved in the m6A modification of small nuclear RNAs (snRNAs), which affects the splicing of pre-mRNAs by snRNAs (Pendleton et al., 2017). Second, m6A modification is also involved in regulating selective splicing (AS) events. m6A signals are enriched in alternative introns and exons in mouse embryonic stem cells, suggesting that they may be associated with RNA splicing (Wei et al., 2021; Tassinari et al., 2023). M6A modification also affects the recruitment of splicing factors through interactions with RNA-binding proteins (RBPs), thereby regulating the splicing patterns of specific genes (Zhu Z. M. et al., 2023). In cancer, abnormal m6A splicing further promotes the survival of tumor cells and contributes to chemotherapy resistance (Wu et al., 2023).

### 2.2 RNA translation

Studies have shown that the 5′UTR of mRNA contains m6A-modified sites that are directly bound by eIF3, which recruits the 43S ribosomal complex to initiate translation in a cap-independent mode. Recent reports indicate that fragile X intellectual disability protein (FMRP) is another m6A-interacting factor that regulates the stability of m6A-containing mRNAs via YTHDF2 (Zhang et al., 2018). m6A modifications regulate mRNA translation through a variety of mechanisms (He and He, 2021).

Recognition of proteins plays a key role in mRNA translation. These genes include YTHDF1, YTHDF3, YTHDC2, and FMR1. YTHDF1 binds to the m6A site around the stop codon, which does not change the overall methylated mRNA level but affects the binding of m6A-tagged mRNA to ribosomes, interacting with eIF3 and recruiting the 43S ribosome complex to promote translation (Wang et al., 2015; Sendinc and Shi, 2023). YTHDC2 bridges the interaction between m6A-containing mRNAs and ribosomes to facilitate translation initiation and extension, thereby increasing mRNA translation efficiency (Kretschmer et al., 2018). YTHDF1 and YTHDF3 act synergistically, with YTHDF3 inducing m6A-modified mRNA transcriptional flow to YTHDF1 to increase translation efficiency (Shi et al., 2017). IGF2BPs have also been reported to promote mRNA stability by preventing mRNA degradation or promoting mRNA storage during stress conditions. thereby facilitating their translation (Huang et al., 2018). METTL3 plays a critical role in inducing mRNA translation by interacting with the eukaryotic translation initiation factor 3 h subunit (eIF3h), mediating mRNA circularization and promoting the translation of numerous oncogenic mRNAs (Choe et al., 2018; Shi et al., 2019). Similarly, FMRP is an RNA-binding protein associated with m6A modification. FMRP acts as a m6A recognition protein that specifically recognizes and binds to m6A-modified mRNAs. FMRP fly homolog FMR1 interacts with the m6A reader YTHDF to inhibit translation of transcripts that regulate axonal growth (Worpenberg et al., 2021). YTHDF1, which binds to ribosomal proteins, promotes translation of its RNA targets. FMRP regulates this mechanism by sequestering YTHDF1 away from the ribosome. Upon neuronal stimulation, FMRP undergoes phosphorylation, leading to the release of YTHDF1 and subsequent translation upregulation (Zou et al., 2023a). These mechanisms collectively form a complex network of m6A modifications involved in the regulation of mRNA translation, finely tuning gene expression and influencing cellular functions and physiological processes in organisms (Edupuganti et al., 2017).

### 2.3 RNA stability and degradation

Decay is the final stage of mRNA metabolism, and the regulation of RNA stability is a key step in controlling the dynamic balance of RNA metabolism and gene expression, which is important for the regulation of cellular functions (Du et al., 2016). m6A increases mRNA decay and destabilizes mRNA by interacting with mRNA through specific binding proteins, regulating cellular degradation mechanisms, or removing stabilizing proteins (Wang et al., 2015; Deng et al., 2018).

YTHDF proteins are major cytoplasmic m6A-binding proteins that have been reported to correlate with the stability of m6A-containing mRNAs and to regulate mRNA degradation pathways. Most of the current studies consistently show that YTHDF2 plays a major role in this pathway. (Zhao et al., 2017). YTHDF2 promotes the rapid degradation of m6A-marked mRNAs mainly through different pathways: 1) When m6A is recognized by YTHDF2, the interaction of YTHDF2 with the CCR4-negative regulatory factor (CCR4-NOT) complex leads to the deadenylation of mRNAs, which is the first step of mRNA degradation. Deadenylated mRNA is more readily recognized by exonucleases and endonucleases, resulting in destabilization and further degradation (Collart, 2016; Du et al., 2016). 2) If the YTHDF2-bound mRNA contains a heat-responsive protein 12 (HRSP12)-binding site, mRNA degradation is initiated preferentially through a ribonucleolytic cleavage reaction mediated by ribonuclease P and ribonuclease MRP (RNaseP/MRP) complexes (Park et al., 2019; Boo and Kim, 2020). In addition to YTHDF2, other proteins containing the YTH structural domain are involved in mRNA degradation. For example, YTHDF1, YTHDF2, and YTHDF3 have overlapping subsets of target transcripts that destabilize mRNAs(Hsu et al., 2017). IGF2BP is a multifunctional reader protein that prevents mRNA degradation by stabilizing target transcripts (Huang et al., 2018). m6A modification plays a crucial role in the regulation of gene expression, which not only affects the translation efficiency of mRNAs but also affects the stability and degradation rate of mRNAs, which in turn finely regulate protein synthesis in the cell (Ma et al., 2019). Our in-depth research indicates that depleting all YTHDF proteins boosts P-body formation, enhancing overall RNA stability, a process not strictly dependent on m6A modification. This suggests YTHDF proteins have complex roles in globally regulating RNA longevity. Their function depends not only on their unique attributes but also on the cellular environment and other regulatory mechanisms (Zou et al., 2023b).

m6A modification regulates RNA stability, degradation, and translation efficiency, playing a key role in gene expression (Kretschmer et al., 2018; Li H. et al., 2022). This epigenetic transcriptional regulation is crucial in normal physiological processes and significantly impacts pathological states (Zhang L. et al., 2024). Notably, in cancer therapy, abnormal m6A modification regulation is closely related to drug resistance (Chen C. et al., 2021; Wang C. et al., 2023). Next, we will explore how m6A modification affects drug resistance in gastrointestinal tumors, analyze its molecular mechanisms, and offer new perspectives and strategies for future cancer therapy.

## 3 Mechanisms of drug resistance associated with m6A RNA modifications in GI tumors

Cancer drug resistance is a major obstacle to cancer treatment and an important reason for poor patient prognosis and treatment failure (Hangauer et al., 2017; Adamson et al., 2023). In recent years, with increasing epigenetic studies, increasing evidence has shown that m6A modifications play important roles in cancer drug resistance (Ordway et al., 2020; Shi et al., 2023). The dynamic regulation of m6A modification is closely related to prognosis, therapeutic efficacy, resistance to chemotherapy, targeted therapy, and immunotherapy in various GI tumors. Therefore, exploring the molecular mechanisms of m6A modification in cancer drug resistance could provide potential targets and new therapeutic strategies for future cancer therapy (Zhu D. H. et al., 2024) (Table 2).

**TABLE 2:** Mechanisms of m6A RNA modifications in the context of chemotherapy resistance in digestive system cancers.

Type	Drug	Gene	m6A regulation	Expression alteration	Role	Downstream targets	Mechanisms	References
GC	Cisplatin	IGF2BP1/METTL3	reader/writer	Upregulation	Oncogene	ABL	IGF2BP1/METTL3/ABL/APAF1/Cytc/caspase-9/3	Wang et al. (2022)
GC	Cisplatin	METTL3/YTHDC1	writer/reader	Upregulation	Oncogene	FAM120A	METTL3/YTHDC1/FAM120A/SLC7A11	Niu et al. (2024)
GC	Cisplatin	METTL3	writer	Upregulation	Oncogene	ARF6	β-elemene/METTL3/ARF6	Song et al. (2024)
GC	Oxaliplatin	METTL3/YTHDF1	writer/reader	Upregulation	Oncogene	PARP1	METTL3/YTHDF1/PARP1	Li et al. (2022a)
GC	Cisplatin	METTL14	writer	Upregulation	Oncogene	CircUGGT2	METTL14/CircUGGT2/miR-186-3p/MAP3K9	Chen et al. (2024a)
GC	Oxaliplatin	WTAP	writer	Upregulation	Oncogene	TGF-β	WTAP/TGF-β	Liu and Da (2023)
GC	Oxaliplatin	KIAA1429	writer	Upregulation	Oncogene	FOXM1	KIAA1429/FOXM1	Tang et al. (2023a)
GC	5-FU	FTO	eraser	Upregulation	Oncogene	CDKAL1	FTO/CDKAL1	Liu et al. (2023c)
GC	Cisplatin	FTO/YTHDF2	eraser/reader	Upregulation	Oncogene	ULK1	FTO/YTHDF2/ULK1	Zhang et al. (2022)
GC	5-FU	hnRNPA2B1	reader	Upregulation	Oncogene	NEAT1	hnRNPA2B1/NEAT1/Wnt/β-catenin	Wang et al. (2024b)
GC	Cisplatin	IGF2BP2	reader	Upregulation	Oncogene	CSF2	IGF2BP2/CSF2/Notch1	Ji et al. (2023)
GC	Cisplatin	METTL3	writer	Upregulation	Oncogene	LINC00942	METTL3/LINC00942/MSI2/C-Myc/DNMT3a	Zhu et al. (2022), Zhu et al. (2023a)
GC	Cisplatin	METTL3	writer	Upregulation	Oncogene	ARHGAP5-AS1	METTL3/ARHGAP5-AS1/ARHGAP5	Zhu et al. (2019)
PC	Gemcitabine	METTL3	writer	Upregulation	Oncogene	DDX23	METTL3/DDX23/PI3K/Akt	Taketo et al. (2018), Lin et al. (2023)
PC	Gemcitabine	METTL14	writer	Upregulation	Oncogene	TGF-β2	METTL14/TGF-β2/PI3K-Akt Signaling/SREBF1	Ma et al. (2024)
PC	Gemcitabine	FTO	eraser	Upregulation	Oncogene	KDM5B	FTO/KDM5B/DLG1/YAP1/SMAD4	Wang et al. (2024a)
PC	Gemcitabine	ALKBH5/IGF2BP1	eraser/reader	Upregulation	Oncogene	SH3BP5-AS1	ALKBH5/IGF2BP1/SH3BP5-AS1/miR-139-5p/CTBP1/Wnt	Lin et al. (2022a)
PC	Gemcitabine	IGF2BP1	reader	Upregulation	Oncogene	EP300	CMTM6/IGF2BP1/EP300	Zhu et al. (2024a)
HCC	Sorafenib	METTL3/YTHDF2	writer/reader	Downregulation	Anti-oncogene	LINC01273	METTL3/YTHDF2/LINC01273/miR-600	Kong et al. (2022)
HCC	Sorafenib	METTL3	writer	Upregulation	Oncogene	SORE	METTL3/SORE/Wnt/miRNA/β-catenin	Xu et al. (2020)
HCC	Sorafenib	METTL3/YTHDF1	writer/reader	Downregulation	Anti-oncogene	FOXO3	METTL3/YTHDF1/FOXO3	Lin et al. (2020)
HCC	Sorafenib	hnRNPA2B1	reader	Upregulation	Oncogene	NEAT1	hnRNPA2B1/NEAT1/Wnt/β-catenin	Wang et al. (2024b)
HCC	Apatinib	METTL3	writer	Upregulation	Oncogene	p53	METTL3/p53	Ke et al. (2022)
HCC	Regorafenib	KIAA1429	writer	Upregulation	Oncogene	CCR9	KIAA1429/CCR9	Lv et al. (2024)
HCC	Regorafenib	METTL14	writer	Downregulation	Anti-oncogene	CHOP	METTL14/CHOP	Pan et al. (2024a)
CRC	Oxaliplatin	METTL3	writer	Upregulation	Oncogene	TRAF5	METTL3/TRAF5	Lan et al. (2021)
CRC	Oxaliplatin/Cisplatin	YTHDF1	reader	Upregulation	Oncogene	GLS1	YTHDF1/GLS1	Chen et al. (2021a), Lin et al. (2024)
CRC	5-FU/Oxaliplatin	METTL3	writer	Upregulation	Oncogene	Sec62	METTL3/Sec62/Wnt/β-catenin	Liu et al. (2021a)
CRC	5-FU	METTL3	writer	Upregulation	Oncogene	circ-0000677	METTL3/circ-0000677/TRAP1	Liu et al. (2022b), Kang et al. (2024)
CRC	5-FU	YTHDC2	reader	Downregulation	Anti-oncogene	LIMK1	YTHDC2/LIMK1/eIF2α	Chen et al. (2023a)
CRC	5-FU	METTL14/YTHDC2	writer/reader	Upregulation	Oncogene	pri-miR-17/miR-17-5p	METTL14/YTHDC2/pri-miR-17/miR-17-5p/MFN2	Sun et al. (2023)
CRC	5-FU	FTO/YTHDF2	eraser/reader	Upregulation	Oncogene	SIVA1	FTO/YTHDF2/SIVA1	Zhu et al. (2024b)
CRC	Cetuximab	hnRNPA2B1	reader	Upregulation	Oncogene	MIR100HG	hnRNPA2B1/MIR100HG/TCF7L2/Wnt	Liu et al. (2022c)
CRC	Cetuximab	IGF2BP3/METTL14	writer	Upregulation	Oncogene	EGFR	IGF2BP3/METTL14/EGFR	Chen et al. (2023b)
CRC	Cisplatin	IGF2BP3	reader	Upregulation	Oncogene	ABCB1	IGF2BP3/ABCB1	Yang et al. (2021)
ES	Cisplatin	ALKBH5	eraser	Upregulation	Oncogene	CASC8/hnRNPL	ALKBH5/CASC8/hnRNPL/Bcl2/caspase3	Wu et al. (2022)
ES	—	METTL3	writer	Upregulation	Oncogene	DGCR8/miR-20a-5p	METTL3/DGCR8/miR-20a-5p/NFIC	Liang et al. (2021)
ES	—	METTL14	writer	Downregulation	Anti-oncogene	miR-99a-5p	METTL14/miR-99a-5p/TRIB2	Liu et al. (2021b)

### 3.1 Mechanism by which m6A RNA modifications mediate drug resistance in GC

GC is the fifth most common malignancy and the third leading cause of cancer-related death worldwide (Smyth et al., 2020; Park and Herrero, 2021; Siegel et al., 2021). Cisplatin (DDP) is the first-line chemotherapy for late-stage GC. However, drug resistance and unavoidable severe toxicity can lead to chemotherapy failure and poor prognosis. The specific role of m6A and potential mechanisms of DDP resistance in GC remain unclear (Ajani et al., 2022; Gao et al., 2023).

Studies have shown that IGF2BP1 recognizes METTL3-mediated m6A modifications on ABL tyrosine kinases (ABLs) and maintains ABL stability, resulting in increased ABL levels. The increased expression of ABL enables its interaction with apoptotic protease-activating factor 1 (APAF1) and competitively blocks the interaction between APAF1 and cytochrome c (Cytc), thereby inhibiting the intrinsic apoptotic pathway, blocking apoptosome assembly and caspase-9/3 activation, and resulting in the resistance of GC cells to cell death. These findings suggest that ABL may be a potential therapeutic target for clinical GC patients (Wang et al., 2022). METTL3 also mediated m6A modification, and YTHDC1 promoted FAM120A stability and significantly increased the expression level of FAM120A. FAM120A binds to SLC7A11 mRNA and enhances its stability, thereby suppressing ferroptosis and promoting cisplatin (DDP) resistance. These findings suggest that targeting FAM120A to overcome cisplatin resistance in GC is a potentially effective strategy. In addition (Niu et al., 2024), METTL3 enhances DDP drug resistance and promotes GC progression partly through m6A-dependent regulation of ADP ribosylation factor 6 (ARF6) expression. In contrast, β-elemene reduces the m6A modification of ARF6 by inhibiting the expression of METTL3, thereby downregulating the expression level of ARF6 and ultimately reversing drug resistance (Song et al., 2024). METTL3 also enhances the stability of polyadenosine diphosphate ribose polymerase 1 (ARP1) mRNA by recruiting YTHDF1, and PARP1 promotes resistance to oxaliplatin (OXA) in CD133+ GC stem cells, enhancing their function by increasing the activity of the base excision repair pathway (Li H. B. et al., 2022).

Other methyltransferases can also induce drug resistance, such as METTL14, which can regulate CircUGGT2 through m6A-dependent modifications, promoting cell proliferation, metastasis, and the development of DDP resistance in GC by adsorbing miR-186-3p and upregulating mitogen-activated protein kinase kinase 9 (MAP3K9) (Chen X. Y. et al., 2024). WTAP is an oncogene that is overexpressed in GC cells, and a significant increase in WTAP expression in GC is closely associated with poor prognosis in GC patients. Studies have shown that WTAP can mediate the upregulation of transforming factor-β (TGF-β) to increase the stability of TGF-β mRNA and promote epithelial‒mesenchymal transition (EMT) in GC cells, and it also promotes chemoresistance and radioresistance in GC cells (Liu and Da, 2023). KIAA1429 promotes OXA resistance in GC cells by promoting FOXM1 mRNA stability (Tang Q. et al., 2023).

The expression of the demethylases FTO and CDKAL1 was upregulated in GC cells and tissues. CDKAL1 is a downstream target gene regulated by FTO through m6A modification. FTO enhances the proliferative capacity of GC cells and induces mitochondrial fusion by promoting the expression of CDKAL1, ultimately leading to the resistance of GC cells to chemotherapy (Liu Y. et al., 2023). In addition, FTO regulated YTHDF2-associated ULK1 expression in GC cells to promote autophagy-induced cisplatin resistance (Zhang et al., 2022). m6A-binding protein hnRNPA2B1 expression is elevated in GC cells and tissues and is positively correlated with poor prognosis, especially in patients treated with 5-fluorouracil (5-FU). hnRNPA2B1 interacts with and stabilizes NEAT1 to activate the Wnt/β-catenin pathway, maintain stemness and aggravate chemotherapy resistance in GC (Wang J. et al., 2024).

**FIGURE 2:**
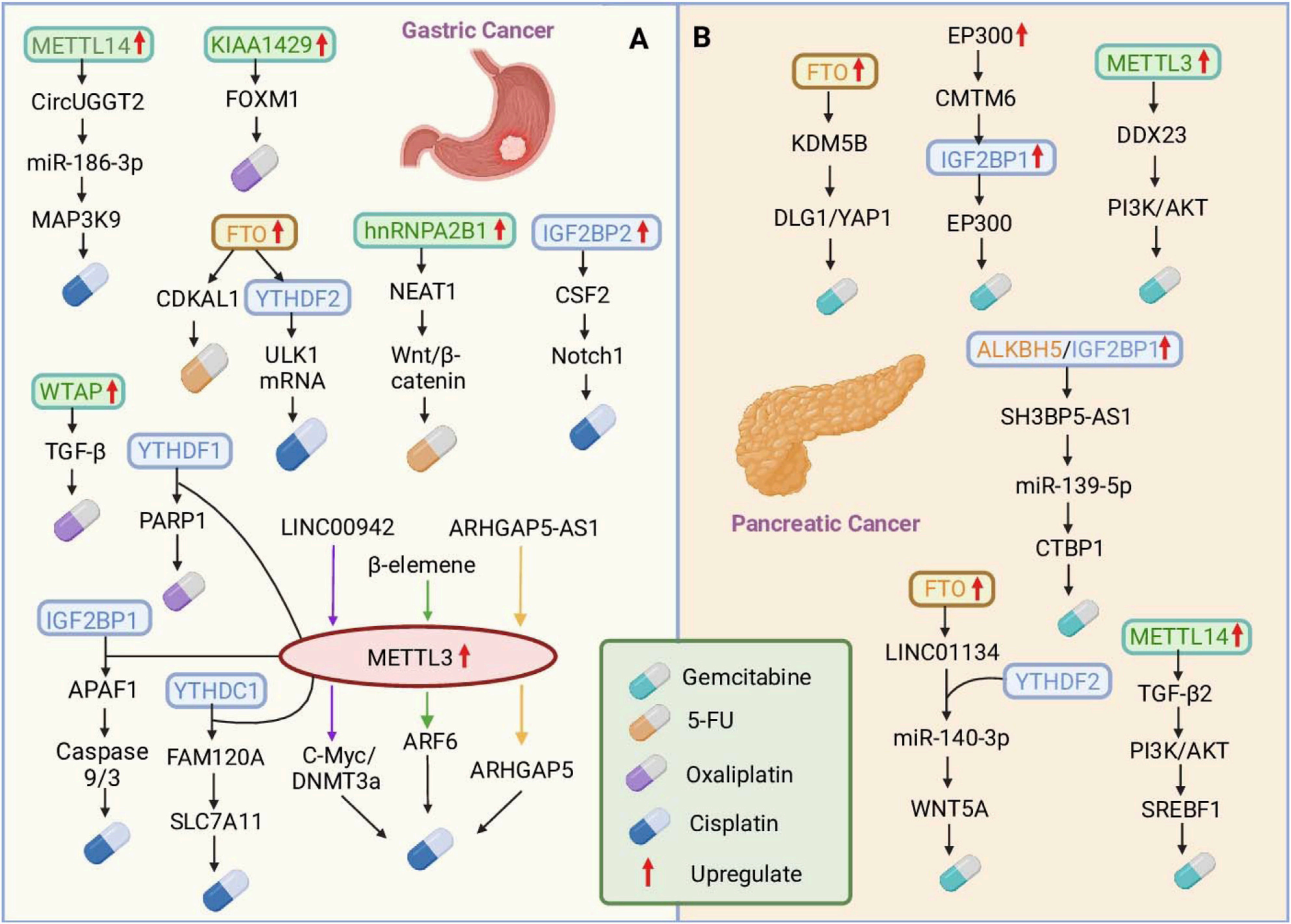
The roles of m6A RNA modifications in mediating chemotherapy resistance in gastric and pancreatic cancers. **(A)** In gastric cancer (GC), dysregulated RNA methylation modulates various pathways to induce resistance to cisplatin (DDP), 5-fluorouracil (5-FU), and oxaliplatin (OXA). The key mechanisms include METTL3-mediated m6A modification, which enhances ARF6 and PARP1 mRNA stability, and hnRNPA2B1, which stabilizes the NEAT1 lncRNA, increasing 5-FU resistance. **(B)** In pancreatic cancer (PC), m6A modification drives gemcitabine (GEM) resistance. METTL3-mediated m6A methylation stabilizes DDX23 mRNA. FTO stabilizes KDM5B expression and activates DLG1/YAP1 signaling, whereas LINC01134, which is regulated by FTO and YTHDF2, sponges miR-140-3p to activate WNT5A and promote resistance. These findings highlight the complex interplay between RNA modification enzymes and downstream pathways, providing potential therapeutic targets to overcome chemotherapy resistance in digestive system cancers.

The TME also contributes to drug resistance (Jin and Jin, 2020; Xiao and Yu, 2021; Xu et al., 2024). The TME induces an increase in IGF2BP2 in mesenchymal stem cells (MSCs), which binds to and enhances the stability of colony-stimulating factor 2 (CSF2) mRNA, and upregulated CSF2 negatively regulates the Notch signaling pathway in MSCs by inducing Notch1 ubiquitination to promote GC progression (Ji et al., 2023). Chemotherapy resistance remains the greatest obstacle to cancer treatment because of many genetic and epigenetic alterations. lncRNAs are emerging players that contribute to cancer onset and progression. lncRNAs regulate a variety of biological processes, including proliferation, metastasis, metabolism, and drug resistance, in cancer. Studies have revealed that the lncRNA LINC00942 is markedly upregulated in GC cells and is strongly linked with poor prognosis. The stability of C-Myc mRNA is enhanced by LINCNC00942 in a m6A-dependent manner (Zhu et al., 2022). In addition, LINC00942 promoted METTL3-mediated m6A modification to stabilize DNA methyltransferase 3a (DNMT3a) mRNA (Zhu L. et al., 2023). Another lncRNA, ARHGAP5-AS1, was upregulated in GC and correlated with poor prognosis. ARHGAP5-AS1 enhances ARHGAP5 mRNA stability by recruiting METTL3 and modifying ARHGAP5 mRNA. All three mechanisms enhance DDP resistance in GC (Zhu et al., 2019) (Figure 2A).

### 3.2 Mechanism by which m6A RNA modifications mediate drug resistance in PDAC

PC is a highly aggressive and deadly cancer (Siegel et al., 2021), and pancreatic ductal adenocarcinoma (PDAC) is the most common histologic type and has the worst survival of all human cancers (Chen D. et al., 2022). Patients with PDAC often develop locally late-stage or metastatic disease at the time of diagnosis and are usually not candidates for radical resection (Traub et al., 2021). Although the efficacy of gemcitabine (GEM), the standard first-line chemotherapy drug for treating PDAC, is remarkable at the initial stage, most patients have a poor prognosis due to the emergence of acquired resistance. In recent years, studies have revealed that m6A modification plays an essential role in the pathogenesis of PDAC, not only in terms of tumor survival and proliferation but also in the context of chemotherapy resistance (Mizrahi et al., 2020; Tempero et al., 2021).

Studies have shown that the methylation transferase METTL3 is markedly upregulated in GEM-resistant PDAC cells and regulates the mRNA expression of the downstream target DDX23 through m6A modification. DDX23 overexpression in PDAC is correlated with poor prognosis, activates the PI3K/Akt signaling pathway, and enhances the proliferation and migration of cancer cells as well as chemotherapy resistance (Taketo et al., 2018; Lin et al., 2023). In addition, TGFβ2 is stabilized at the posttranscriptional level by METTL14-mediated m6A modification, which promotes high TGFβ2 expression. Upregulated TGFβ2 promotes the expression of sterol regulatory element-binding protein 1 (SREBF1) by activating the PI3K–AKT signaling pathway. SREBF1, a key regulator of lipid metabolism, further upregulates the expression of downstream lipid synthetases, leading to the accumulation of intracellular lipids. Reprogramming of lipid metabolism alters the chemical properties of PDAC cells and enhances resistance to GEM (Ma et al., 2024).

In PDAC, m6A-binding proteins also play key roles in regulating drug resistance. FTO stabilizes the expression of KDM5B through demethylation, and KDM5B acts as a coregulator by activating the downstream DLG1/YAP1 pathway. KDM5B promotes tumorigenesis in the context of SMAD4 loss by reprogramming the accumulation of lipids in PDAC, as well as resistance to the chemotherapy drug GEM (Wang Y. et al., 2024). FTO also promotes the expression of LINC01134 mRNA in PDAC by stabilizing it, while LINC01134 directly binds to YTHDF2, which competitively binds to miR-140-3p to deregulate the inhibitory effect of miR-140-3p on WNT5A, thereby activating the WNT pathway and enhancing the resistance of patients to GEM (Lu et al., 2023). In addition, SH3BP5-AS1 is markedly upregulated in GEM-resistant PCs and predicts a poor prognosis. SH3BP5-AS1 was negatively correlated with the level of ALKBH5, and its stability was mediated by the ALKBH5/IGF2BP1-mediated modification of m6A. The deletion of SH3BP5-AS1 reduced the migration and invasion of PC cells and increased the susceptibility of PCs to GEM. Upregulated SH3BP5-AS1 reduced its inhibition of C-terminal binding protein 1 (CTBP1) by adsorbing miR-139-5p and upregulated CTBP1 expression by activating the Wnt signaling pathway, promoting GEM resistance in PC (Lin H. et al., 2022).

CKLF-like MARVEL transmembrane domain-containing protein 6 (CMTM6) is upregulated in GEM-resistant PDAC, and high CMTM6 expression is correlated with poor prognosis. EP300-mediated modification of H3K27ac activates CMTM6 transcription. CMTM6 inhibits IGF2BP1 degradation in PDAC cells by reducing its polyubiquitylation. IGF2BP1 sustains the expression of EP300 and MYC mRNAs via m6A modification. This creates a positive feedback loop involving EP300, CMTM6, IGF2BP1, and EP300 (mRNA), which strengthens tumor stemness and promotes drug resistance in PDAC. Studies have shown that targeting m6A modifications may be a potential strategy to improve the efficacy of pancreatic cancer therapy, although the role of m6A modifications in pancreatic cancer drug resistance still requires further study (Zhu Y. Q. et al., 2024) (Figure 2B).

### 3.3 Mechanism by which m6A RNA modifications mediate drug resistance in HCC

Primary liver cancer, including the hepatocellular carcinoma (HCC) and cholangiocarcinoma (CCA) subtypes, is the sixth most prevalent cancer and the fourth leading cause of cancer-related death worldwide (Barsouk et al., 2020). HCC is usually detected in the late stage and is not surgically treatable; molecularly targeted therapies and immunotherapies are the keys to the treatment of late-stage HCC (Ye et al., 2024), of which sorafenib (SOR) is recognized as the first molecularly targeted drug used for clinical HCC treatment; however, drug resistance has become a major obstacle to treatment (Kong et al., 2021). Recent research has revealed that m6A regulatory factors are strongly associated with the progression of HCC and have potential as therapeutic targets (Chan et al., 2024).

**FIGURE 3:**
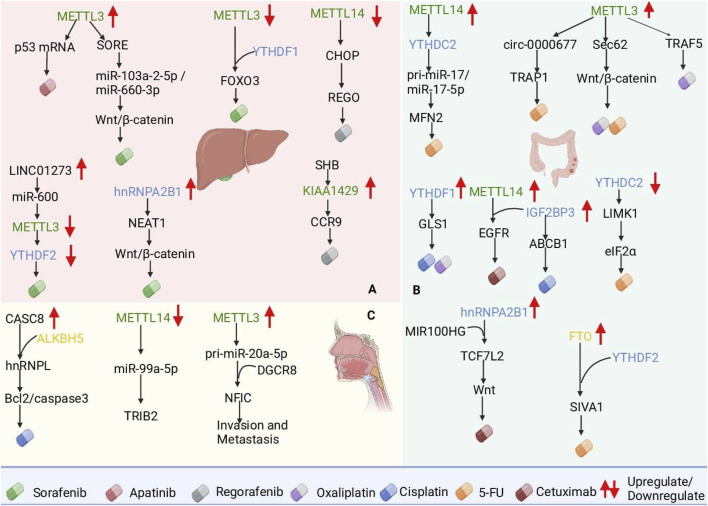
Roles of m6A RNA modifications in mediating chemotherapy resistance in hepatocellular carcinoma (HCC), colorectal cancer (CRC), and esophageal cancer (ESCA). **(A)** In HCC, METTL3 modulates sorafenib resistance by stabilizing circRNA-SORE and reducing FOXO3 mRNA stability. **(B)** In CRC, METTL3 and FTO regulate Sec62 and SIVA1 mRNA, promoting resistance to 5-FU and oxaliplatin. **(C)** In ESCA, ALKBH5-mediated CASC8 upregulation stabilizes hnRNPL, activating the Bcl2/caspase3 pathway, whereas METTL14 loss promotes radioresistance via TRIB2. These findings underscore m6A regulators as potential therapeutic targets to overcome resistance in gastrointestinal cancers.

Studies have shown that LINC01273 is markedly upregulated in HCC and SOR-resistant tissues, increasing the stability of miR-600 and thus METTL3 mRNA inhibition, thereby leading to METTL3 downregulation and SOR resistance. In addition, METTL3 increased HCC LINC01273 m6A levels and decreased the stability of LINC01273 recognition of YTHDF2 (Kong et al., 2022). In particular, METTL3 is usually defined as an oncogene and is upregulated in most tumors; for example, METTL3 upregulation increases the level of m6A modification of circRNA-SORE, which segregates oncogenic miRNAs (e.g., miR-103a-2-5p and miR-660-3p), thereby activating the Wnt/β-catenin pathway and inducing sorafenib resistance (Xu et al., 2020). However, METTL3 expression was significantly downregulated in SOR-resistant HCC cells. The downregulation of METTL3 expression decreases the stability of FOXO3 mRNA, which is regulated in a YTHDF1-dependent manner, and thus promotes SOR resistance in HCC. Studies have shown that METTL3 has a dual role, and its internal mechanism deserves further study (Lin et al., 2020). Furthermore, hnRNPA2B1 interacts with and stabilizes the lncRNA NEAT1, which contributes to the activation of the Wnt/β-catenin pathway and increases chemoresistance in HCC (Wang J. et al., 2024). HCC is resistant not only to SOR but also to apatinib (APTN) and regorafenib (REGO). Studies have shown that the upregulation of METTL3, which increases p53 mRNA instability and decreases its expression level, increases HCC resistance to APTN (Ke et al., 2022). Small HBV surface antigen (SHB) promotes HCC progression and REGO resistance through KIAA1429-mediated modification of C-C chemokine receptor 9 (CCR9) m6A (Lv et al., 2024). In addition, downregulation of METTL14 reduced m6A modification of C/EBP homologous protein (CHOP) mRNA and decreased CHOP protein levels, and low CHOP levels reduced the apoptosis-inducing effect of REGO on tumor cells, increasing tumor resistance to REGO (Pan Y. et al., 2024) (Figure 3A).

### 3.4 Mechanism by which m6A RNA modifications mediate drug resistance in CRC

CRC is the third most common cancer worldwide. Radical surgical resection is the first choice of treatment, and radiotherapy, as a conventional postoperative treatment strategy, can significantly increase patient survival rates (Liu H. et al., 2022). OXA, a third-generation platinum-based drug, is widely used as a first-line chemotherapeutic agent for the treatment of CRC, and the development of resistance to chemotherapeutic agents by CRC is a major problem for CRC patients. In OXA-resistant patients, m6A levels are elevated in CRC tissues (Zhao et al., 2022).

Both METTL3 and YTHDF1 overexpression resulted in OXA resistance in CRC patients, in which METTL3 induced resistance to OXA through receptor-associated factor 5 (TRAF5)-mediated necroptosis (Lan et al., 2021), and YTHDF1 induced resistance to OXA through the upregulation of glutaminase 1 (GLS1) expression and induced resistance to OXA and DDP (Chen P. et al., 2021; Lin et al., 2024). In addition to OXA, 5-FU is also used as a first-line drug for the treatment of CRC. Studies have shown that METTL3 promotes cancer cell stemness and survival and enhances resistance to the chemotherapeutic drugs 5-FU and OXA by upregulating Sec62 and activating the Wnt/β-catenin signaling pathway (Liu X. et al., 2021). METTL3 also promotes CRC resistance to 5-FU through circ-0000677 and tumor necrosis factor (TNF) receptor-associated protein 1 (TRAP1) (Liu Q. et al., 2022; Kang et al., 2024). In addition, the downregulation of YTHDC2 increased the stability and expression of LIM kinase 1 (LIMK1) mRNA, and LIMK1 overexpression promoted eIF2α phosphorylation (Chen L. et al., 2023). By reducing YTHDC2 binding to pri-miR-17, METTL14 promotes the stability of pri-miR-17 mRNA and increases miR-17-5p expression, and the overexpression of miR-17-5p inhibits mitochondrial fusion protein 2 (MFN2), which ultimately leads to 5-FU resistance (Sun et al., 2023). FTO upregulation inhibits YTHDF2-mediated apoptosis-inducing factor 1 (SIVA1) degradation and reduces SIVA1 expression by removing m6A modifications on SIVA1 mRNA, thereby increasing antiapoptotic effects and resistance to 5-FU in CRC cells. CRC patients are also able to develop resistance to the targeted therapeutic drug cetuximab (Zhu D. H. et al., 2024). Studies have revealed that MIR100HG and hnRNPA2B1 overexpression increases TCF7L2 mRNA stability and activates Wnt signaling, leading to resistance to cetuximab (Liu et al., 2022c). In addition, IGF2BP3 was upregulated in CRC tissues, and elevated levels of IGF2BP3 predicted a poor prognosis. IGF2BP3 promotes the stability and translation of epidermal growth factor receptor (EGFR) mRNA and further activates the EGFR pathway by cooperating with METTL14 to increase cetuximab resistance in CRC cells (Chen L. J. et al., 2023). IGF2BP3 upregulates ABCB1 gene expression by binding to RNA with m6A modifications, which in turn enhances drug efflux and leads to cisplatin (DDP) resistance in colorectal adenocarcinoma (HCT8) cells (Yang et al., 2021) (Figure 3B).

### 3.5 Mechanism by which m6A RNA modifications mediate drug resistance in ESCA

Esophageal cancer (ESCA) is one of the most common malignant tumors worldwide (Abnet et al., 2018). Esophagectomy is the first choice for early-stage ESCA, and late-stage ESCA has a poor prognosis (Fan et al., 2021). Esophageal squamous cell carcinoma (ESCC) is the major subtype of ESCA in developing countries (Zhang M. et al., 2024). m6A methylation is a critical epigenetic modification that plays a role in both the physiological and pathological processes of tumors. However, its function in esophageal cancer remains unclear, but recent studies have shown that, as an early diagnostic marker and therapeutic target, m6A modification has significant potential in the treatment of esophageal cancer (Zhao et al., 2021).

Studies have shown that ALKBH5-mediated demethylation of m6A leads to the overexpression of cancer susceptibility candidate 8 (CASC8) in ESCC and that CASC8 upregulation predicts poor prognosis in ESCC patients. CASC8 reduces the DDP sensitivity of ESCC cells and promotes ESCC tumor growth *in vivo*. CASC8 interacts with and inhibits the polyubiquitination of heterogeneous nuclear ribonucleoprotein L (hnRNPL), thereby stabilizing hnRNPL protein levels and activating the Bcl2/caspase3 pathway. This promotes cell proliferation and DDP resistance in ESCC. CASC8, a key node of this pathway, may be a potential target for ESCC therapy (Wu et al., 2022). In addition, METTL3 has been identified as an important promoter of cancer progression and is highly expressed in esophageal cancer. METTL3 promotes m6A modification and the binding of DiGeorge syndrome critical region gene 8 (DGCR8) to miR-20a-5p, which further increases miR-20a-5p expression and inhibits nuclear factor IC (NFIC) transcription, thereby promoting EMT, invasion and migration (Liang et al., 2021). Downregulation of METTL14 expression in ESCC significantly enhances the resistance of ESCC to radiation therapy by hindering the maturation process of miR-99a-5p and deregulating the inhibitory effect of miR-99a-5p on TRIB2 expression (Liu Z. et al., 2021). Cytoplasmic activation/proliferation-associated protein-1 (Caprin-1), whose expression is significantly elevated in both ESCA tumor tissues and cell lines, is associated with poor prognosis in ESCA patients. Silencing Caprin-1 inhibited glycolysis to suppress ESCA cell proliferation and downregulated the expression of METTL3 and WTAP to delay ESCA progression. Therefore, Caprin-1 may serve as a prognostic marker and potential therapeutic target for ESCA. The above mechanism of action suggests that targeting m6A modifications may become a new strategy to improve the therapeutic efficacy of esophageal cancer treatment (Gao et al., 2023) (Figure 3C).

## 4 m6A in immunotherapy resistance

In addition to resistance to chemotherapy and targeted therapies, some resistance to immunotherapy has been shown in cancer patients. Immune checkpoint blockade (ICB) therapy is an effective cancer therapy, and immune checkpoint inhibitor (ICI) therapy, such as programmed cell death protein 1/programmed cell death ligand 1 (PD-1/PD-L1) inhibitors and cytotoxic T lymphocyte antigen 4 (CTLA-4) inhibitors, has been successful in different cancers (Ribas and Wolchok, 2018; Fares et al., 2019; Mizrahi and Pant, 2020; Tong et al., 2021). m6A plays a key role in regulating immunotherapy resistance. In HCC, METTL3 inhibition enhances the efficacy of anti-PD-1 therapy in a m6A-YTHDF2-dependent manner (Wu et al., 2024). In CRC, downregulation of poly C-binding protein 1 (PCIF1) increases the sensitivity of CRC tumors to anti-PD-1 therapy by regulating the Fos-Tgf-β and Stat1/Ifitm3-IFN-γ axes (Wang L. et al., 2023). In PDAC, overexpression of ALKBH5 increases Wnt inhibitor 1 (WIF-1) transcription via demethylation, suppressing the Wnt pathway and modulating tumor immune evasion (Tang et al., 2020). In GC, IGF2BP1 is overexpressed, enhancing PD-L1 mRNA stability, which boosts PD-L1 expression and allows tumor cells to evade immune system attacks (Jiang et al., 2024; Tang et al., 2024). Studies have shown that low expression of YTHDF1 may be a marker for the strong response of gastrointestinal cancers to ICB treatment. These findings suggest that YTHDF1 may be a feasible target to improve the efficacy of ICB in the treatment of gastrointestinal cancers (Chen S. et al., 2022). m6A-modified circQSOX1 promotes glycolysis and immune escape in CRC by regulating the miR-326/miR-330-5p/PGAM1 axis and inhibits the response to CTLA-4 antibody immunotherapy in CRC patients, leading to immunotherapy resistance (Liu et al., 2022d). These findings demonstrate that m6A modification influences immune resistance in gastrointestinal tumors through diverse mechanisms, such as immune checkpoint modulation, immune cell infiltration and functional effects, and tumor microenvironment alterations. These insights highlight potential targets and a theoretical basis for developing novel immune therapy strategies.

Increasing evidence consistently links alterations in m6A regulatory proteins and overall m6A modification patterns to gastrointestinal cancer development and progression (Zhu Y. Q. et al., 2024). We reviewed the biological functions and potential molecular mechanisms of m6A RNA methylation, its regulators, and recent advances in downstream target genes. Thus, a deeper understanding of the role of m6A in drug resistance in GI tumors can aid in the development of novel therapeutic strategies, overcome drug resistance, and improve therapeutic efficacy (Liu H. et al., 2022; Liu N. et al., 2023).

## 5 Conclusion and outlook

RNA modification, which plays a key role in tumorigenesis and drug resistance, is an important area of research on epigenetic regulation. m6A modification, the most common form of chemical modification in eukaryotic mRNAs, has received much attention in recent years. m6A modification is regulated by three core regulatory proteins, namely, methyltransferase proteins responsible for the addition of the m6A modification (e.g., METTL3, METTL14), demethylases responsible for the removal of the m6A modification (e.g., FTO, ALKBH5), and binding proteins that recognize the m6A tag (e.g., YTHDF1/2/3, YTHDC1). Together, these regulatory proteins constitute a dynamic regulatory network for m6A modification, which affects gene expression and cellular function in several ways, including influencing RNA splicing, translation, stability, and degradation, thus influencing cancer development.

In GI tumors, m6A modification abnormalities are thought to be closely related to tumor development as well as drug resistance. In this paper we further explored the important role of m6A modifications in the drug resistance mechanism of digestive system tumors (e.g., gastric, colorectal, hepatic, pancreatic, and esophageal cancers) and analyzed the correlations between m6A levels, patient prognosis, and drug resistance in detail. For example, METTL3 affects several specific lncRNAs or circRNAs (e.g., circQSOX1) and influences the expression of glycolysis-related genes, thereby increasing the resistance of tumor cells to the chemotherapeutic drug 5-FU. In addition, abnormal upregulation of FTO reduces the expression level of certain proapoptotic genes (e.g., SIVA1) by removing m6A modifications, thereby inhibiting apoptosis and enhancing chemotherapy resistance. m6A modifications have also been shown to play important roles in immunotherapy. For example, by regulating the expression of immune inhibitory molecules such as PD-L1, m6A modification can enhance tumor immune escape and weaken the therapeutic efficacy of immune checkpoint inhibitors (e.g., anti-PD-1/PD-L1 antibodies). These mechanisms reveal the broad impact of m6A modifications on drug resistance in tumor cells and provide important targets for the development of new therapeutic strategies.

Although the role of m6A modification in cancer has been widely researched, the specific mechanism of its role in drug resistance still has many unanswered questions. Future studies should focus on the multidimensional resolution of the dynamic regulation of m6A modification, especially its different mechanisms of action in different types of digestive tract tumors. In addition, m6A modification affects the biological behavior and drug resistance of tumor cells by regulating RNA fate. The development of specific drugs targeting m6A-regulated proteins is important for overcoming tumor drug resistance. For example, inhibitors of FTO or METTL3 have shown significant therapeutic potential in clinical studies. Combining m6A-targeted drugs with existing chemotherapies, targeted therapies, or immunotherapies is expected to significantly improve cancer treatment outcomes.

In conclusion, the study of m6A modification not only expands our understanding of RNA regulation but also provides new perspectives and ideas for the exploration of drug resistance mechanisms in cancer. An in-depth analysis of the role of m6A modification in the drug resistance mechanism of digestive system cancers and the development of targeted therapeutics will help overcome the drug resistance problem in cancer treatment and improve the therapeutic efficacy and survival rate of patients.
